# Optimum Drug Combinations for the Sedation of Growing Boars Prior to Castration

**DOI:** 10.3390/ani7080061

**Published:** 2017-08-10

**Authors:** Heidi S. Lehmann, Dominique Blache, Eleanor Drynan, Pema Tshewang, David J. C. Blignaut, Gabrielle C. Musk

**Affiliations:** 1 School of Veterinary and Life Sciences, Murdoch University, Perth 6150, Australia; E.Drynan@murdoch.edu.au (E.D.); D.Blignaut@murdoch.edu.au (D.J.C.B.); 2 School of Agriculture and Environment, University of Western Australia, Perth 6009, Australia; dominique.blache@uwa.edu.au (D.B.); 21190515@student.uwa.edu.au (P.T.); 3 Animal Care Services, University of Western Australia, Perth 6009, Australia; gabrielle.musk@uwa.edu.au

**Keywords:** pigs, sedation, castration, detomidine, midazolam, butorphanol, morphine

## Abstract

**Simple Summary:**

Pigs are notoriously challenging patients. They are difficult to handle so the use of predictable and safe sedation techniques is required for husbandry and surgical procedures. Various combinations of sedative and analgesic drugs have been previously investigated in this species, though the combination of midazolam and detomidine with either butorphanol or morphine has not been reported for sedation in pigs. The use of these combinations was investigated in the context of adequate sedation to allow castration of boars with the aid of local anaesthetic infiltration. The combination of midazolam, detomidine with butorphanol provided a more reliable sedation combination than that including morphine. It is proposed that this combination of drugs would be useful for sedation during painful husbandry procedures in pigs.

**Abstract:**

Juvenile male pigs were sedated for castration. Eight five-month old boars were sedated twice (two weeks apart) with a combination of detomidine (0.1 mg/kg), midazolam (0.2 mg/kg) and either butorphanol (0.2 mg/kg) (Group MDB, n = 8) or morphine (0.2 mg/kg) (Group MDM, n = 8) intramuscularly. The boars were positioned in lateral recumbency and lidocaine (200 mg total) was injected into the testicle and scrotal skin. Castration of a single testicle was performed on two occasions. Sedation and reaction (to positioning and surgery) scores, pulse rate, respiratory rate, haemoglobin oxygen saturation, body temperature, arterial blood gas parameters and the times to immobility and then recovery were recorded. Atipamezole was administered if spontaneous recovery was not evident within 60 min of sedative administration. Data were compared with either a paired-sample *t*-test or a Wilcoxon-Signed Rank Test. There was no difference in sedation score, body temperature, respiratory rate and haemoglobin oxygen saturation between MDB and MDM. Mild hypoxaemia was noted in both groups. There was less reaction to castration after MDB. The pulse rate was higher after MDM sedation. The times to immobility and then recovery were similar. The combination of MDB provided more reliable sedation than MDM. MDB may be useful for sedation for short procedures in pigs, though oxygen supplementation is recommended to avoid hypoxaemia.

## 1. Introduction

Pigs are typically difficult animals to handle, and therefore to examine and treat by veterinary personnel, in both research and clinical settings. Sedative and premedication drugs are consequently often required to enable the implementation of these procedures and are commonly administered by intramuscular injection [1]. The ideal combination of drugs for administration by this route to a pig should be small in volume, safe for both animals and personnel, provide predictable sedation, facilitate a smooth recovery, be specifically antagonised and be readily accessible to veterinary practitioners. The requirement for such sedative or premedication drug combinations for husbandry procedures such as castration, tusk or claw trimming is increasing in veterinary practice. Moreover, pigs are often treated outside of hospital locations with limited access to appropriate facilities and trained personnel. Several drug combinations are reported for such use and minimising injectate volume is a common aim given the difficulty of restraining the animal.

Dissociative anaesthetic drugs like ketamine and tiletamine are often included in pig sedation protocols, including in combination with sedative agents azaperone, detomidine, medetomidine, dexmedetomidine and butorphanol [2,3,4,5]. Utilising these potent dissociative anaesthetic agents aids in reducing the volume of drug for administration, often described as a limitation for practical swine sedation [3,6]. However, there are a number of undesirable features of sedation and anaesthesia produced by dissociative agents and these include unreliable sedation, seizures and rough recoveries [2,7]. Recovery from sedation with these agents may be stressful for the animal involved because of emergent delirium induced by *N*-methyl-d-aspartate (NMDA) receptor and anticholinergic receptor interactions, and no specific reversal agent is available [8].

The short-acting neurosteroid anaesthetic alfaxalone has also been used as the primary agent, often in combination with sedative or tranquiliser agents, for sedation in pigs [3,9]. While the sedation produced by combinations of alfaxalone and sedative agents dexmedetomidine or diazepam were described as “deep” and of a better quality than those incorporating ketamine or alfaxalone alone, it is clearly indicated by both sets of authors that animal size beyond small (approximately 20 kg bodyweight) is a particular limiting factor. Due to the potency of the current formulation of alfaxalone (1% solution in cyclodextrin, Jurox, Australia), the injectate volume of these combinations is considerable (>10 mL for pigs up to 22 kg bodyweight), limiting practical usefulness to small pigs [3,9]. The identification of drug combinations for reliable sedation in pigs, combinations that do not include ketamine or alfaxalone and provide reduced restrictions on the bodyweight of the animal and the use of antagonist agents, is therefore clinically important.

Combinations of drugs for sedation in pigs [3,4,6,9] include α-2 adrenoreceptor agonists, benzodiazepines and opioids. A limitation to using these agents is the volume of the injection when using standard commercial formulations with fixed concentrations. The greatest variability in potency occurs amongst the α-2 adrenoreceptor agonist agents. The α-2 adrenoreceptor agonist agents medetomidine, dexmedetomidine and xylazine have been trialed previously in various combinations or as a sole agent for sedation of pigs [9,10,11]. Pigs are particularly resistant to α-2 adrenoreceptor agonists, and as such higher doses, and by extension higher injection volumes, are required for sedation [12]. Thus, more potent α-2 adrenoreceptor agonist agents such as detomidine have potential to provide sedation and analgesia whilst reducing the volume of drug for injection [2,13]. A high-potency α-2 adrenoreceptor agonist agent typically used in veterinary practice is detomidine at 1% (10 mg/mL), commonly used for sedation in equine patients. Inclusion of detomidine in mixtures with fixed concentration benzodiazepine and opioid agents may permit the volume of sedation injectate to be minimised. Atipamezole has been used to antagonise the sedative effects of medetomidine in pigs with no adverse effects noted [4,14]. However it has not yet been reported in pigs for the antagonism of detomidine.

Previous reports suggest that benzodiazepine agents midazolam or diazepam may provide reasonable sedation in pigs when used alone [15] or in combination with other agents including ketamine [7,16] and alfaxalone [9]. Midazolam is preferable to diazepam for the use of intramuscular injection due to the water-solubility of the commercial formulation, high bioavailability and insensitivity to ultraviolet light compared to diazepam. Benzodiazepines can be reversed by the specific antagonist flumazenil and sufficient reversal of midazolam-induced sedation in pigs is reported [17]. Flumazenil has a shorter duration of action than midazolam or diazepam so residual sedation may occur. Accordingly, close monitoring and subsequent repeated dosing of flumazenil may be required to optimise recovery from benzodiazepine sedation. 

Morphine is a pure µ-opioid receptor agonist that has not been extensively evaluated for sedation in pigs. It is commonly used for analgesia and sedation in a range of other species including dogs [18,19], cats [20] and horses [21]. Butorphanol, a κ-opioid receptor agonist and µ-opioid receptor antagonist, has frequently been reported in sedative drug combinations for pigs, with good results [2,4,10,11].

The aim of this study was to investigate the combination of midazolam and detomidine with either butorphanol or morphine for sedation in pigs. These drug combinations were chosen to evaluate a low-volume sedative and analgesic drug combination for the provision of reliable and predictable sedation suitable for short clinical procedures in pigs, such as castration. It was hypothesised that pigs receiving detomidine and midazolam with butorphanol would be more profoundly sedated than those receiving detomidine and midazolam with morphine. 

## 2. Materials and Methods 

### 2.1. Ethics

The study was approved by the Animal Ethics Committees at the University of Western Australia (RA/3/100/1451) and Murdoch University (N2833/16) and was performed in accordance with the Australian Code for the Care and Use of Animals for Scientific Purposes [22].

### 2.2. Animals

Eight healthy five-month old entire male bacon pigs (Landrace × Large White × Duroc) from a commercial property were obtained and housed at the Large Animal Facility of the University of Western Australia for two weeks prior to the study. The pigs were housed in communal raised floor pens (4 × 5 m) in temperature controlled (22 ± 2 °C) rooms with 12:12 h light:dark cycle. The pigs were fed a maintenance diet (Pig Grower, West Feeds Pty. Ltd., Perth, Australia) with fresh pumpkin and apples and had free access to tap water. Environmental enrichment was provided by music during the day, various toys for play and daily human interaction. The pigs were clinically normal prior to sedation and surgery when examined by a veterinarian.

Sedation with a combination of midazolam and detomidine with either butorphanol (group MDB, n = 8) or morphine (group MDM, n = 8) was utilised. In a cross-over, block-randomised study design, each of the eight pigs were allocated to each of the two treatment groups on two occasions for a unilateral castration each time, separated by two weeks. This arrangement gave a total, for each treatment group, of eight subjects. Surgical castration of a single testicle was performed on two separate days, two weeks apart. On the first surgery day four pigs received MDB for sedation and four pigs received MDM for sedation. Two weeks later the other testicle was surgically removed and the alternative sedation protocol was used for each pig.

### 2.3. Sedation

The pigs were fasted overnight (12 h) and weighed in the transport crate (1.5 × 0.4 m) immediately prior to sedation. Midazolam (0.2 mg/kg; Symbion, Docklands, Australia), detomidine (0.1 mg/kg; Detomo Vet, Ceva Animal Health, Glenorie, Australia) and either butorphanol (0.2 mg/kg; Torbugesic, Zoetis, Rhodes, Australia) or morphine (0.2 mg/kg; Sigma, Castle Hill, Australia) was combined in a single syringe prior to administration. The doses were selected from previous publications of sedative combinations in pigs [10,13,23]. The drug doses were calculated, prepared and administered by intramuscular injection in the trapezius muscles by a single anaesthetist (G.C.M.). After ten minutes the pigs were moved from the crate and positioned on a surgical table in the lateral recumbency. Their position was determined by their position in the crate i.e., if they were in left lateral recumbency in the crate then this position was assumed when they were moved to the surgery table. During the second sedation and castration the alternative lateral position was utilised. 

A tight-fitting facemask was placed over the nose and mouth and 2 L/min of medical air (21% oxygen) was supplied via a circle breathing system connected to an anaesthetic machine (Datex-Ohmeda GE AS/3, Madison, WI, USA). Lidocaine (10 mL, 2% lignocaine hydrochloride, Ilium Lignocaine 20, Troy laboratories, Sydney, Australia) was injected into the non-dependent testis and the subcutaneous skin of the scrotum. Five minutes later surgical castration was performed. Three independent blinded observers (E.D., D.J.C.B., D.B.) scored sedation at two time points; ten minutes following administration of sedation and then throughout the castration procedure from when the pig was moved onto the surgery table until an arterial blood gas sample was collected. All observers and the author were blinded to the treatment until after the experiment was completed. 

Firstly, a score out of ten was attributed to the degree of sedation (sedation score) before the pigs were removed from the transport crate (ten minutes after injection) (Table 1). There were no stimuli applied that would influence the sedation score; Second, each observer gave a score out of four for reaction (reaction score), based upon the response to being moved from the crate to the table, the injection of lidocaine and subsequent castration (Table 2). For data analysis, the sedation and reaction scores for each animal were the sum of the three observers’ scores. At the end of surgery, the pigs were placed in a recovery pen (1.2 × 1.8 m). If no attempts to stand were made within 60 min of the administration of sedative drugs, atipamezole (30 μg/kg, 5 mg/mL, Ilium Atipamezole, Troy Laboratories, Sydney, Australia) was administered intramuscularly. 

### 2.4. Sedation and Recovery Kinetics

The behaviour of the pigs was recorded using mounted camcorders. Video recordings were made prior to injection of the sedative mixture while the pig was in the transport crate, during transfer to the surgery table, during transfer from the surgery table to the recovery pen and during the final transfer to the communal pen. From the video footage, the time to kneel down (folding forelimb under the body), to lie down (all limbs folded and head on the ground) and to become immobile (no movement for at least a minute taken as the start of sedation) was recorded following the injection of sedative drugs. The times to first movement (swaying head), to extending the forelimbs (extending the forelimb forward and lifting front of the body), to first attempt to stand on four limbs (all four feet on the ground and body lifted) and to stand for one minute were recorded during the recovery phase. These data are expressed relative to the time of injection of the sedative mixture and relative to each other.

### 2.5. Surgery

An open castration technique was used for castration of the boars and the scrotal incision was closed to reduce interference by fellow animals in the post-operative period. Following preparation of the skin for aseptic surgery, the non-dependent testis was digitally immobilised in the scrotum. A single longitudinal incision, parallel to the median raphe, was made through the scrotal skin, subcutaneous tissue and *tunica vaginalis.* The testis, epididymis and part of the spermatic cord were identified and isolated. The spermatic cord and vessels were ligated. The spermatic cord was incised and the testis, epididymis and distal part of the spermatic cord were removed. The *tunica vaginalis* was closed with a continuous suture pattern with absorbable suture material (Vicryl, Ethicon, Inc., Johnson & Johnson Medical Pty. Ltd., North Ryde, Australia). The scrotal skin was closed with a continuous intradermal suture pattern using absorbable suture material (Vicryl, Ethicon, Inc., Johnson & Johnson Medical Pty. Ltd., North Ryde, Australia).

### 2.6. Monitoring

Physiological measurements (heart rate, respiratory rate and haemoglobin oxygen saturation) were measured continuously using a multiparameter monitor (SurgiVet V9203, Surgivet, Ferntree Gully, Australia) and recorded every five minutes. Rectal temperature was measured before and after surgery. A single arterial blood sample was collected into a pre-heparinised syringe (RAPIDLyte, Siemens Healthcare, Bayswater, Australia) from an auricular artery at the completion of surgery. Topical local anaesthetic cream (EMLA 5%, AstraZeneca, Macquarie Park, Australia) had been applied to this site as soon as the animal had been positioned on the surgical table. Arterial blood gas analysis was performed immediately following collection (RAPIDPoint 500, Siemens Healthcare, Bayswater, Australia). 

### 2.7. Post-Operative Care

The pigs were monitored twice a day for the two-week period between sedation and surgery and for two weeks after the second procedure. Ethograms, motor balance tests and serial salivary cortisol concentrations were assessed in this period (data not shown). Well-being was determined by observing and scoring mucous membrane colour and capillary refill time, demeanour, respiratory rate and effort, gait, appetite, water intake, presence of secretions from the eyes, nose and surgical wound, urination and defaecation habits (data not shown). Rescue analgesia was meloxicam (0.5 mg/kg subcutaneously) and/or morphine (0.5 mg/kg intramuscularly) administered if a specialist veterinary anaesthetist (G.C.M.) deemed it necessary in conjunction with the use of a composite pain score adapted from a previous report [24]. At the end of the study period the pigs were euthanised with intravenous pentobarbitone.

### 2.8. Data Analysis

Data were assessed for normality using a Shapiro-Wilk test. Parametric variables were compared using a paired-samples *t*-test, and nonparametric assessment was completed using a Wilcoxon-Signed Rank Test. All data analysis was completed using SPSS software (Version 22.0.0.0, IBM, New York, NY, USA). Parametric data are presented as mean ± SD and non-parametric data are presented as median (range). *p* < 0.05 was considered significant.

## 3. Results

All eight animals completed both blocks of the study and no animals required rescue analgesia post-operatively. In addition to the well-being assessment completed in the post-operative period, composite pain-scores, ethograms, motor balance tests and serial salivary cortisol concentrations were documented (data not shown).

**Figure 1:**
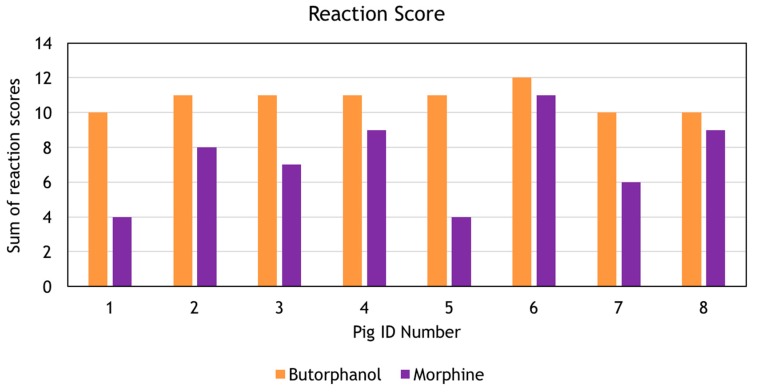
Reaction scores to surgery (sum of individual observers scores) of each pig by the three observers. MDB = midazolam, detomidine and butorphanol, MDM = midazolam, detomidine and morphine.

Before removal from the transport crate the sedation score for all animals was 30. The reaction score was significantly higher in the MDB group (10.75 ± 0.71) than the MDM group (7.25 ± 2.49), *p* = 0.003 (Figure 1). There was no difference in the weight, volume of drug for injection, duration of the procedure, body temperature, mean respiratory rate and mean haemoglobin oxygen saturation (SpO_2_) between the groups. The SpO_2_ could not be measured in one animal in the MDM group due to movement artefact evident on the plethysmograph. This movement contributed to a lower reaction score in this animal. Body temperature at the end of surgery was not recorded for one animal in the MDB group. The mean pulse rate during the procedure was higher in the MDM group (Table 3). Arterial blood could not be collected from one animal in the MDB group and three animals in the MDM group. The arterial pH was higher in the MDM group but all other arterial blood gas variables were similar (Table 4). No additional doses of sedative drugs were given. 

There was no difference between the two sedation protocols for any of the timing of the behaviours recorded during the sedation phase (Table 5). There was no difference between the two protocols in the number of times the pigs tried to stand up after the injection of sedative drugs (Table 5). One pig receiving MDB struggled more than the other pigs and tried to stand 12 times while the other pigs tried one to six times. After the administration of MDM the pigs started to move approximately 19 min earlier, and to extend their forelimbs about 16 min earlier than those receiving MDB, but there was no difference between the two sedation protocols in the time of the first attempt to stand (72 ± 23 min) and finally time to standing (Table 5). All pigs were standing 84 ± 6.0 min after the injection of the sedative mixture. Atipamezole was administered on 13 (out of 16) occasions 60 min after the administration of sedation; it was not administered to three animals in the MDM group. All animals were eating within two hours of sedation being administered.

## 4. Discussion

The current study demonstrated that a combination of midazolam, detomidine and butorphanol provided better quality sedation for castration, with local anaesthesia, of growing boars when compared to a combination of midazolam, detomidine and morphine. The “reaction score” constructed for this study provided a simple descriptive scale with which to score the quality of sedation when a noxious stimulus was applied.

Detomidine was chosen as the α-2 adrenoreceptor agonist for the current study because of the relatively high concentration of the formulation (1% solution). Previous reports of the use of detomidine in pigs are in combination with butorphanol and ketamine [2,13]. In both studies between 10 and 33% of animals were not adequately sedated, as opposed to the current study where all animals were satisfactorily sedated for castration after receiving MDB. This difference between the combinations of drugs used in this study, and those reported elsewhere, provides evidence that ketamine may produce unreliable sedation and that midazolam may contribute to better quality sedation than ketamine. While the efficacy of atipamezole for antagonism of detomidine was not a specific aim of the current study, this drug was successfully used to facilitate recovery from sedation in the 13 animals that received it. Given not all animals required the use of atipamezole to facilitate recovery, it may be inferred that the pharmacokinetics of the sedative agents varied amongst individuals. The use of atipamezole after 60 min was an arbitrary time-point, based on empirical evidence.

Several antagonist agents are used successfully to reverse opioid sedation and side-effects. Naloxone is the opioid antagonist most commonly reported for use in pigs [27], though reversal of butorphanol has shown to be less complete than that of morphine, purportedly due to receptor specificity differences. The longer acting agent naltrexone is used in wildlife species [28,29], with little reported use in domestic species.

The present data demonstrates that the combination of midazolam, detomidine and butorphanol provides reliable and adequate sedation in pigs for castration with adjunctive use of local anaesthesia. Pigs receiving MDM had lower reaction scores than those that received MDB. This reactivity prevented the collection of arterial blood samples so limited the physiological evaluation of the MDM drug combination. In addition to the decreased sedation during castration, animals that received MDM recovered sooner and fewer of these animals required atipamezole, compared to those receiving MDB. It may be that the dose of morphine was inadequate to allow meaningful comparison between the two groups and further investigations into higher doses of morphine for use in combination with midazolam and detomidine may be warranted. Additionally, the increased pulse rate in the MDM group may indicate that morphine provided less analgesia than butorphanol in this context. It must be noted that the reversal of morphine by the antagonist naloxone is complete. However much higher doses are required for reversal of butorphanol [30]. At the doses used in this study, the depth and duration of sedation was more appropriate for castration using MDB.

The sedation score used in this study demonstrated that an isolated observation of a sedation-only score was not able to differentiate between sedation protocols used for castration [25,26]. Few reports exist on species-specific sedation scores used in pigs. Similar to the current study, a composite score assessing recumbency, reaction to environmental stimuli along with response to procedural techniques gave the most robust assessment [7]. The summation of sedation and reaction scores in the current study allowed each blinded assessor to be weighted equally, and therefore accounted for inter-assessor variation.

Assessment of arterial blood gas showed that although the pH was higher during sedation with MDM, this difference was not considered clinically relevant. The absence of samples from some animals did not appear to affect the spread of data, though it must be noted that the pH results from both groups are not normally distributed so a larger study population may be required to decrease this Type I error. No other significant differences were noted in the other arterial blood gas variables. Both groups were mildly hypoxaemic [31], and thus oxygen supplementation is warranted when using these protocols. The use of an FiO_2_ of 21% was selected to mimic ambulatory clinical conditions. The findings of this study suggest the utilisation of oxygen supplementation is advisable.

There were several limitations to this study. The injectate volume was not standardised as in other studies [3,9]. However, the volumes used were not significantly different between treatment groups. All volumes were between 3.2 and 5.1 mL and thus easy to inject rapidly. Standardisation of the volume of drug for injection is adopted in other reports assessing the reaction to the injection itself. The current study did not assess this parameter as an outcome, thus standardisation of the drug doses was considered a sound approach. The scores for sedation and reaction used here have not been validated and require further evaluation to determine how appropriate they are for this context. However, as previously noted, there are few reported scoring systems used in pigs, and as such, further work in this area is warranted. The doses of the opioid agents were selected empirically from previous reports [10] and a single dose of each was evaluated. Further investigations using multiple doses in multiple treatment groups may be prudent. 

Additionally, specific assessment of analgesic effects of the drug combinations may also be useful, though it is acknowledged that the assessment of acute pain is difficult in pigs and has uncommonly been reported in previous studies [32]. Only very recently have researchers examined specific nociceptive assessment methods in conscious pigs during acutely painful stimulation, utilising nociceptive threshold testing and facial grimace scoring [33,34,35,36]. The use of such equipment and analysis would be a suitable next step to the current project. Furthermore the pharmacokinetics of these drug combinations in similar animals to those used in the current study would aid the development of appropriate dosing regimes and intervals for practical application, particularly the determination of suitable dosing beyond empirical evidence. The pharmacokinetics of such agents may also aid in the criteria for when to use the antagonist agents, often an expensive component in clinical practice.

## 5. Conclusions

In conclusion, the combination of detomidine, midazolam and butorphanol provides adequate sedation for pigs undergoing castration with the adjunctive use of local anaesthesia. The use of morphine instead of butorphanol in the same combination is not as reliable in this clinical context. The supplementation of oxygen by a facemask is recommended.

## Figures and Tables

**Table 1:** Sedation score used to evaluate sedation ten minutes following the injection of sedative drugs. Adapted from Kastner [25] and Murdoch [26].

Score	Spontaneous Activity	Reaction to Approach	Head Drop	Ataxia	Sternal Recumbency
**0**	**+++**	**+++**	**-**	**-**	**-**
**1**	**++**	**+++**	**-**	**-**	**-**
**2**	**+**	**+++**	**-**	**-**	**-**
**3**	**+**	**++**	**-**	**-**	**-**
**4**	**+**	**+**	**-**	**-**	**-**
**5**	**+**	**+**	**+**	**-**	**-**
**6**	**+**	**+**	**+**	**+**	**-**
**7**	**-**	**+**	**+**	**+**	**-**
**8**	**-**	**+**	**++**	**++**	**-**
**9**	**-**	**-**	**+++**	**+++**	**-**
**10**	**-**	**-**	**+++**	**+++**	**+**

**Table 2:** Reaction score used to evaluate reaction throughout the castration procedure from when the pig was moved onto the surgery table until an arterial blood gas sample was collected. The scores indicated the overall response to being moved, having lidocaine injected, being castrated and having the arterial blood sample taken.

Score	Observation
1	Marked reaction to restraint and/or procedure
2	Moderate reaction to restraint and/or procedure
3	Minimal reaction to restraint and/or procedure
4	No reaction to restraint and/or procedure

**Table 3:** Animal and procedural data. MDB = midazolam, detomidine and butorphanol, MDM midazolam, detomidine and morphine, T = temperature. * = *p* < 0.05.

	GROUP		
Parameter	MDB	MDM	*p* Value
Weight (kg)	54.0 ± 8.7	53.6 ± 9.6	0.932
Volume of drug for injection (mL)	3.98 ± 0.51	3.91 ± 0.71	0.569
Duration of procedure (min)	35 ± 3	36 ± 5	0.620
T prior to lidocaine administration (°C)	37.9 ± 0.4	38.1 ± 0.6	0.454
T at the end of surgery (°C)	37.9 ± 0.5	37.8 ± 0.5	0.571
Pulse Rate (bpm)	71 ± 8	76 ± 9 *	0.033
Respiratory Rate (brpm)	33 ± 7	36 ± 5	0.154
SpO_2_ (%)	92.1 (81.0–99.0)	94.8 (82.3–96.3)	0.499

**Table 4:** Arterial blood gas data. MDB = midazolam, detomidine and butorphanol, MDM = midazolam, detomidine and morphine. * = *p* < 0.05.

	GROUP		
Blood Gas Variable	MDB (n = 7)	MDM (n = 5)	*p* Value
pH	7.466 (7.407–7.489)	7.497 (7.441–7.586) *	0.043
PaO_2_ (mmHg)	70.1 ± 13.1	82.9 ± 16.9	0.339
PaCO_2_ (mmHg)	48.1 ± 3.2	42.1 ± 6.0	0.705
HCO_3_^–^ (mmol/L)	34.2 ± 2.8	32.7 ± 3.2	0.617
Lactate (mmol/L)	0.80 (0.60–2.24)	0.64 (0.49–0.98)	0.893
SaO_2_ (%)	93.4 ± 4.5	96.2 ± 2.2	0.703

**Table 5:** Time of observed behaviours during the induction of sedation, and recovery, in pigs injected with a combination of midazolam, detomidine and either butorphanol (MDB) or morphine (MBM). n = 8 per group. # relative to time of administration of sedative drug combination.

Behaviours	MDB	MDM	*p* Value
**Induction of sedation**			
Number of attempts to stand	4.29 ± 1.38	1.14 ± 0.13	0.100
Time to kneel down (min) #	3.81 ± 0.58	3.84 ± 0.58	0.432
Time to lie down (min) #	4.90 ± 0.40	5.32 ± 1.07	0.844
Time to immobility (min) #	8.53 ± 0.75	8.12 ± 1.24	0.757
Interval between kneeling and lying (min)	1.09 ± 0.46	1.48 ± 1.11	1.000
Interval between kneeling and immobility (min)	4.73 ± 0.84	4.28 ± 1.40	0.951
Interval between lying and immobility (min)	3.64 ± 0.53	2.80 ± 0.84	0.463
**Recovery phase**			
Number of attempts to stand	2.63 ± 0.56	1.63 ± 0.26	0.203
Time to first movement (min) #	67.81 ± 2.56	48.81 ± 5.29	0.015
Time to first attempt to extend forelimbs (min) #	69.15 ± 2.52	53.17 ± 5.15	0.035
Time to first attempt to stand on 4 limbs (min) #	72.27 ± 2.18	72.11 ± 5.72	0.978
Time to stand (min) #	88.63 ± 9.25	78.50 ± 7.72	0.204
Interval between first movement and first attempt to extend forelimbs (min)	1.34 ± 0.23	4.36 ± 1.70	0.039
Interval between first movement down and first attempt to stand (min)	4.46 ± 0.69	23.30 ± 3.96	0.008
Interval between first attempt to stand and standing (min)	16.35 ± 9.41	6.39 ± 4.31	0.250
